# Chemical and Genetic Variability of Istrian *Foeniculum vulgare* Wild Populations

**DOI:** 10.3390/plants11172239

**Published:** 2022-08-29

**Authors:** Mitja Križman, Jernej Jakše

**Affiliations:** 1 Laboratory of Food Chemistry, National Institute of Chemistry, Hajdrihova 19, SI-1000 Ljubljana, Slovenia; 2 Agronomy Department, Biotechnical Faculty, University of Ljubljana, Jamnikarjeva 101, SI-1000 Ljubljana, Slovenia

**Keywords:** *Foeniculum vulgare*, densitometry, allele frequency, PCR-RFLP, headspace-gas chromatography, internal transcribed spacer (ITS), *Apiaceae*

## Abstract

Wild *Foeniculum vulgare* populations from the region of Istria have been subjected to a genetic and chemical study. Headspace-gas chromatography analysis of volatile secondary metabolites and PCR-RFLP analysis of the ribosomal DNA internal transcribed spacer region has been chosen to analyze the chemical and genetic traits of single plants, respectively. Large intrapopulation and interpopulation differences have been observed in both chemical profiles and restriction patterns of PCR products. The data from chemical and genetic analyses were pooled and used to assess allele frequencies of three putative genetic loci on individual populations. The pooled allele frequencies were used to determine interpopulation distances for phenogram reconstruction. The combined use of chemical and genetic datasets for genetic variation analysis proved to be a more comprehensive approach for such a study, compared to the use of single datasets, even using such relatively simple analytical tools.

## 1. Introduction

Fennel (*Foeniculum vulgare* Mill.) has been used for centuries in the Mediterranean basin for both culinary and medicinal purposes. Besides its high content of essential oils [1], fennel has proven to be also a valuable source of various phenolic compounds [2] which are gaining in interest due to their antioxidant, radical scavenging, and other beneficial properties [3,4,5,6,7]. Publications on fennel chemical composition have been so far mostly focused on essential oil analyses, with some of them focused on variations in essential oil composition between different populations [8,9].

Phylogenetic studies have been conducted on the *Apiaceae* family [10,11,12,13,14], mostly based on nuclear ribosomal DNA (nrDNA) internal transcribed spacer (ITS) sequences, but the mentioned papers studied only interspecific relationships. Despite its lower informativeness in comparison to sequencing, the polymerase chain-reaction–restriction fragment length polymorphism (PCR-RFLP) technique was successfully employed for various intraspecific phylogenetic studies [15,16,17,18,19,20]. The first known paper (on fennel molecular genetics was published by Bennici et al. [21], wherein genetic variability of *in vitro* regenerated plants was studied, and a high genetic stability was reported. Only in the last decade or so, the molecular genetic aspects of fennel gained interest [22,23,24,25,26,27,28,29,30]. There was even an attempt to draft the fennel genome [31].

It was our attempt to perform both chemotypic and genotypic screening on a large number of fennel plants from different locations in the Istria region, where fennel is found in abundance. Although for chemotypic screening there was no doubt about the use of gas chromatography (GC) as a well-proven technique in this research field [8,9,32,33], the choice of an adequate genetic technique was not unambiguous. Random amplified polymorphic DNA (RAPD) markers were *a priori* avoided, due to their known unreliability when dealing with diploid plant samples [34,35], as in our case. Microsatellite or amplified fragment length polymorphism (AFLP) [22,23] would probably be the molecular markers of choice, due to their information richness. However, the development of such a marker system is usually quite lengthy and expensive. Moreover, even such selective markers like AFLP alone might not give unequivocal results [22]. Thus, we rather opted for an inexpensive, simple, and more straightforward methodology, namely PCR-RFLP, which is known for its reliability and repeatability [15] in combination with the mentioned chemical screening of the fennel essential oil profile. Rahali et al. [36] in their research work already found the benefits of combining the molecular genetic and chemical analysis data, in their case on *Ferula communis*.

Due to the outbreeding nature of fennel [9], we assumed a certain degree of polymorphism among fennel populations. For the same reason, we used the nuclear DNA internal transcribed spacer (nrDNA ITS) locus for PCR-RFLP analysis, rather than plastid or mitochondrial loci, because we wanted to consider any possible gene flows in fennel populations, occurring by means of pollen migration. In addition, PCR-RFLP electrophoretic sample patterns were quantitatively evaluated by densitometry and the obtained data were conveniently used for allele frequency determination of the nrDNA ITS locus. Such an approach enabled us to gather more data in comparison to electrophoretic band scoring alone.

## 2. Results and Discussion

### 2.1. Chemical Analyses

The same sets of fennel samples were used for both chemical and genetic analyses, and were collected during the flowering season. We avoided the use of samples at the ripe stage, because higher levels of secondary metabolites could inhibit PCR of the extracted DNA [37]. Although the chemical profile of the essential oil changes during ripening, especially the terpene profile, our previous studies indicate that the ratio between both phenylpropanoids remains quite constant during this period. Moreover, the occurrence or absence of fenchone during flowering is also indicative of its presence or absence in mature plants (unpublished results). These results, regarding the composition of essential oil (*trans*-anethole, estragole, and fenchone content) are also in agreement with the analytical results published by Marotti et al. [38]. Moreover, the consistency of the phenylpropanoid ratio throughout plant development is not unexpected, because *trans*-anethole and estragole share a common biosynthetic pathway [39]. Therefore, we used the analytical results obtained from flowering plants as a good predictor of their chemotype at the ripe stage. Fennel plants have shown a high chemical heterogeneity, with chemotypes containing virtually only *trans*-anethole or only estragole, and a whole range of ratios between them. Fenchone was present in the majority of plants analyzed, but its absence was not strictly chemotype-dependent. Most of the nine plant populations studied exhibited a high heterogeneity, but mean population values have shown significant differences between populations. The average *erc* and *arc* values are displayed in Table 1, along with their extreme values found in a given population.

The vast majority (over 90%) of all fennel plants assayed contained various amounts of fenchone, a terpene found in the bitter fennel variety (*Foeniculum vulgare* Mill. var. *vulgare*) [40], which is not surprising, because bitter fennel is very common in the Mediterranean area [39]. Thus, only a minority of all plants lacked fenchone, typical of the sweet fennel (*Foeniculum vulgare* Mill. var. *dulce*), a cultivated variety containing prevalently *trans*-anethole [40]. However, with only two exceptions, those plants contained prevalently estragole (*erc* from 1.0 to 0.44), which is not a characteristic of sweet fennel. There were only two cases of plants with low *erc* and consequently high *arc* lacking fenchone, one from each population (Flengi and Plomin).

The sporadic absence of fenchone in fennel plants should be attributed to fenchone biosynthesis as a dominant trait. The non-relatedness between the absence of fenchone and the variability in phenylpropanoid contents observed in such cases indicates a significant number of allelic variants in corresponding genes.

On the other hand, a wide distribution in phenylpropanoid composition observed by us (Figure S1A) and other researchers [8,9] indicates a complex relationship between the enzymes involved in *trans*-anethole and estragole biosynthesis. Although environmental conditions could influence to some extent the phenylpropanoid biosynthesis [9,41], a wide distribution of chemotypes encountered in single populations should be attributed to gene flows between wild (or naturalized) fennel populations, with a possible influence of cultivated sweet fennel populations as well. These suppositions are in accordance with the problems met by Miura et al. [42] in obtaining chemically uniform seed-germinated fennel plants and with the observed cross-breeding ability of fennel [9]. Consequently, there are little possibilities to find chemically homogeneous wild or naturalized fennel populations.

The wide distribution in the profile of phenylpropanoids, and their high content in fennel plants [40], also suggests the existence of a multicopy gene family involved in phenylpropanoid biosynthesis, which led us to consider *erc* and *arc* as good indicators for allele frequencies of the corresponding genes.

On the contrary, besides its presence at the ripe stage, fenchone timewise accumulation pattern was found to be rather hard to predict. As a consequence, the arbitrary approach described in Section 3.2 was employed to assess the number of its putative (single-copy) genes in a plant sample, which were then used to calculate the *fp* allele frequency in a population. The choice for 15% of fenchone content in the essential oil, as a criterion between putative *fp* monoallelic and biallelic plants, was made on the basis of the histogram shown in Figure S1B. From the pooled results of fenchone content from all plants analyzed we deduced, in a Mendelian fashion, the presence of three fennel plant groups: a small group of putative *fa* homozygotes (8.5% of individuals), a larger one of putative *fp* homozygotes (17.6% of individuals) and the largest one of putative heterozygotes (73.9% of individuals). Although such an approach for genetic assessment could be more arguable as in the case of phenylpropanoid composition, it is supported by the distribution of those three groups and allowed us to evaluate this trait in a codominant manner as well.

Despite the observed intra-population heterogeneities, the pooled allele frequency data revealed differences between populations (Table 1). Because of the nature of the derived chemical data, Cavalli–Sforza and Edward’s chord distance [43], which was originally developed to analyze allele frequencies in human populations, was found to be the most adequate dissimilarity coefficient [44].

**Figure 1:**
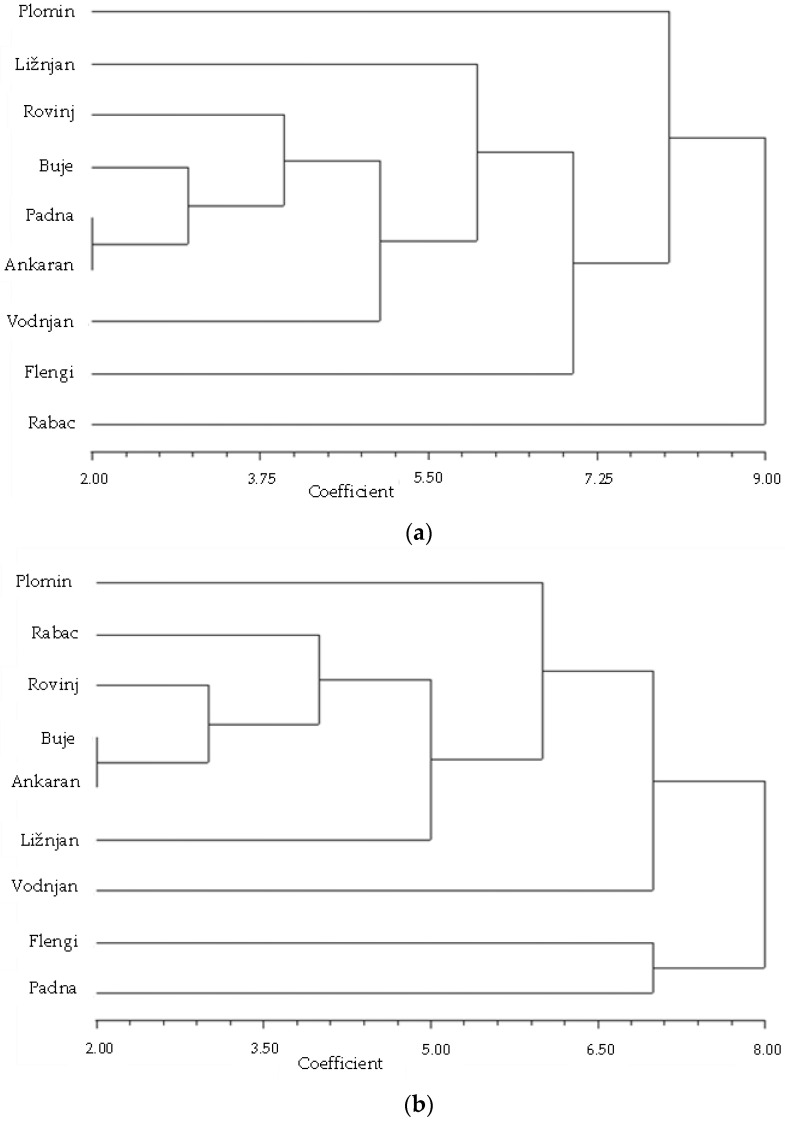
Phenograms of fennel populations reconstructed by using Cavalli–Sforza and Edward’s chord distances [43] and neighbor-joining [45] applied on (**a**) chemical data and (**b**) genetic data. See Section 3.5 for details.

The relationships based on chemical data are depicted in Figure 1a. As is evident, only one tightly spaced pair of populations appear (Ankaran and Padna), whereas the remainder of populations is quite evenly spaced, with no clear clustering pattern present. The outermost outgroup in this case is, as expected, the Rabac population, due to its lowest *erc* frequency. Apparently, the chemical criteria alone could not resolve the relationships between these populations.

### 2.2. PCR-RFLP

**Figure 2:**
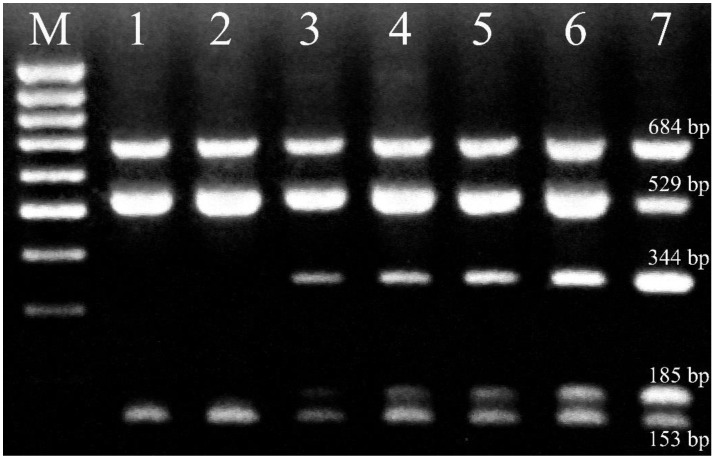
PCR-RFLP electrophoretic patterns of selected samples by using *Msp*I for restriction (1–7). M, DNA 100 bp size standard.

The PCR-RFLP method was optimized to fit the purpose. PCR conditions were optimized in getting the best compromise between the intensity and sharpness of the ITS band, in order to facilitate the densitometric quantitation of electrophoretic gels and to speed up the amplification procedure. *Msp*I endonuclease was selected for large-scale PCR-RFLP analyses because it provided the highest degree of polymorphism among the samples. Two distinct groups of banding patterns occurred by using *Msp*I (Figure 2). In the first group (samples 1 and 2), the restriction yielded two bands of approximately 529 bp and 153 bp in length, indicating the presence of one restriction site. In the second group, another restriction site was also present, located within the 529 bp band, which gave two additional bands of approximately 344 bp and 185 bp in length. However, in both groups, a considerable amount of the ITS band amplificate itself, approximately 684 bp in length was present even after restriction (up to 46% of band area). Prolonged restriction times, increased endonuclease concentrations, or smaller amounts of PCR product digested, did not affect the restriction pattern. Within those two groups of restriction patterns, band area percentages were highly variable between individual plant samples, but highly reproducible in replicate analyses of a given sample. Reproducibility of the PCR-RFLP method was determined on 7 representative samples (Figure 2) in 4 replicates. Average values and standard deviations were calculated from the obtained data (Table 2).

The differences in band intensities observed and their consistent occurrence throughout replicate analyses led us to the use of densitometry instead of simple band scoring. Despite its usefulness, densitometry in electrophoretic gel evaluation is usually used only for a more precise band length determination. There are few papers published where densitometry was employed for a quantitative assessment in DNA gel electrophoresis [16,46,47,48]. Within both groups of banding patterns, a wide distribution of band intensities has been observed among the samples analyzed, without very distinct clusters of samples within a certain group. A plausible explanation for such an even distribution is the high copy number of the ITS locus present within the cluster of ribosomal genes, typically present in several hundred copies per genome in higher eukaryotes [49], and consequently a possible even distribution of putative mutations within this locus. Based on the PCR-RFLP analyses performed by using *Msp*I, up to three allelic forms of the ITS locus in a fennel genome could be identified. The first allele form (*itsA*) lacks a *Msp*I restriction site; thus no restriction products are generated. This form was present in a varying, but considerable amount, in all fennel samples. The second allelic form (*itsB*), carrying one *Msp*I restriction site (generating 529 bp and 153 bp restriction products), was also present in all samples, whereas the third allelic form (*itsC*), carrying another *Msp*I restriction site (generating 344 bp, 185 bp and 153 bp restriction products), was present in about 36% of all samples. The *itsC* frequencies were highly variable among populations (see Table 1). There were probably various mechanisms involved in generating such a variety of proportions between those three allelic forms in fennel. From the evolutionary, long-term standpoint, point mutations and indels are probably important, but on the short-term timescale, fennel cross-breeding tendency and especially chromosome crossing-over should provide the major contribution in population variability, because the ribosomal gene cluster in fennel is located on a single pair of homologous chromosomes [50]. Quantitative densitometry has proven to be useful in unveiling the distribution of allele frequencies, similarly as in the cases of Gürtler et al. [16] and Breen et al. [47].

Similarly, as encountered in chemical composition, PCR-RFLP data displayed the varying degree of heterogeneity within and between fennel populations. For genetic data, the same approach was used as for chemical data. The genetic clustering pattern (Figure 1b) differs greatly from the chemical one (Figure 1a). The former reveals a slightly more defined clustering pattern, but there are still no unambiguous population clusters evidenced. Another significant difference is in the outermost outgroup, in this case represented by a population pair (Flengi and Padna), genetically the most homogeneous, with a complete lack of allele *itsC*. No apparent correlation between both clustering patterns could be deduced. As already discussed in Section 2.1, the genes involved in terpene and phenylpropanoid biosynthesis are probably genetically distant. Similarly, we can also deduce that the ribosomal gene cluster is genetically distant from both groups of biosynthetic genes. Despite the fact that there was a lack of direct correlation between chemical and genetic data, the latter gave us another, independent confirmation of how highly variable this plant species is. This is in accordance with the difficulty found in classifying wild fennel populations into distinct subspecies based only on chemical criteria [41].

### 2.3. Analysis Using Combined Data

In the attempt to obtain a more comprehensive image of relationships between fennel populations, we also combined chemical and genetic data, being both represented as allele frequencies, using the already mentioned analytical approach. Based on the suppositions made in Section 2.1 and Section 2.2, fenchone, phenylpropanoid and ITS allele frequencies could be treated as three independent loci and thus complementary population parameters.

**Figure 3:**
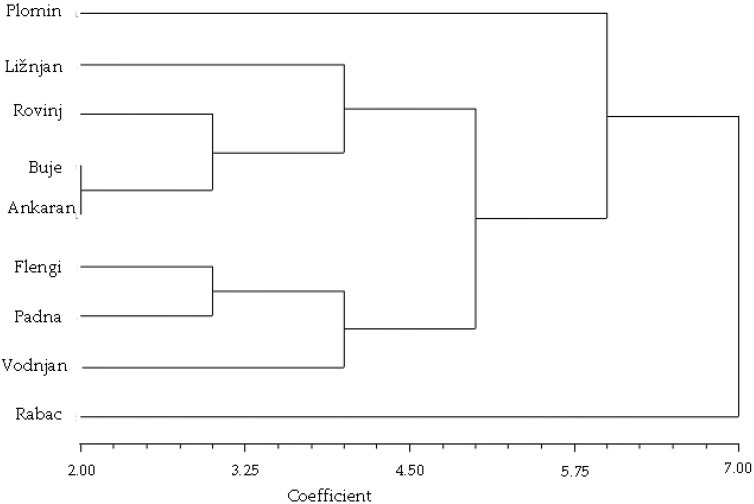
Phenogram of fennel populations reconstructed by Cavalli–Sforza and Edward’s chord distances [43] and neighbor-joining [45] using combined chemical and genetic data.

**Figure 4:**
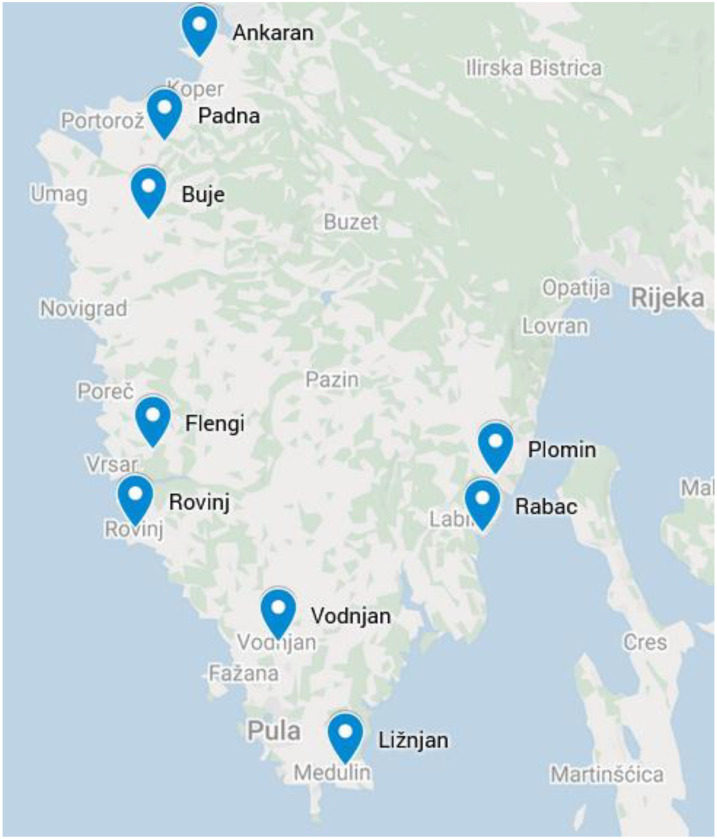
Geographic locations of fennel populations in study in Istria. Retrieved from GoogleMaps.

The clustering pattern in the dendrogram of combined data (Figure 3) differs from both previous ones (Figure 1). There are two distinct clusters and two outgroups present. The larger cluster is represented by the populations found on the Istrian western coast and in its vicinity (Ankaran, Buje and Rovinj), with the exception of one, located at the extreme south, but still facing the Adriatic Sea (Ližnjan). The distances between populations within the cluster are also in agreement with their geographic positions (Figure 4). The *fp* frequencies are quite similar, but a steady decrease in both *erc* and *itsC* frequencies could be observed from north to south. The second cluster is represented by three populations not located on the coastal area (Flengi, Padna and Vodnjan). They differ in *erc* and *fp* frequencies, but there are similarities in the ITS locus. There is a complete lack of the *itsC* allele in two of them (Flengi and Padna), but their common denominator is a slight, but apparent drift in the *itsB* frequency going from north to south as well. The first outgroup (Plomin) is located on the eastern Istrian coast, facing the Kvarner Gulf. This population has a rather low *itsC* frequency, similar to Vodnjan population, while the *fp* and *erc* frequencies are similar to those from Ližnjan and Flengi populations, respectively.

The outermost outgroup (Rabac), located also on the eastern coast, differed substantially from all other populations. The most evident differences were the lowest *erc* and the highest *fp* frequencies among all, whereas its *itsB* and *itsC* frequencies were more typical of the northwestern populations. It also displayed the highest chemical homogeneity. The Rabac population is probably a recently established offspring from a cultivated bitter fennel plantation, without the time needed to become sufficiently cross-pollinated from neighboring populations and consequently diversified. Excluding the Rabac population, a generalized statement about two allele frequency drifts could be made; the *itsC* is decreasing from north to south and from the coast to the inland area, whereas *erc* is decreasing from north to south and from the western to the eastern coast.

To corroborate the observed allelic drifts in Istrian fennel populations, more of the latter should be examined, possibly along with topological, and meteorological data at the time of flowering, with the aim to obtain an objective explanation of such an allelic distribution. Anyway, the employed approach of combining chemical and genetic analysis for plant population studies looks promising, with little effort needed for method development. Consequently, it could be a valuable, high-throughput tool for population data gathering. Another option using this approach, which should be also considered in future work, is a proper sample pooling technique, for a further reduction of experimental efforts.

## 3. Materials and Methods

### 3.1. Plant Material and DNA Extraction

One hundred and fifty-five fennel samples were collected during the flowering period in the Istria region (Slovenia and Croatia) from wild growing populations, on nine different locations: Ankaran, Buje, Flengi, Ližnjan, Padna, Plomin, Rabac, Rovinj, and Vodnjan (Figure 4). Ten to twenty leaf samples from individual plants were collected on each location. The samples were immediately frozen in liquid nitrogen and kept refrigerated (at −20 °C) until needed for analysis.

Template DNA was extracted from leaf samples of individual plants according to a previously published protocol [51]. The extracted DNA was quantified by a fluorometric assay by using a DyNA Quant™ 200 fluorometer and a DNA-specific dye, Hoechst H33258 (Hoefer, Holliston, MA, USA) according to manufacturer’s instructions.

### 3.2. Chemical Analysis

Leaf samples from individual plants were subjected to the analysis of essential oil components by using headspace-gas chromatography as described in our previous paper [32]. The estragole relative content (*erc*) was calculated as the ratio between estragole peak area divided by the sum of peak areas from both fennel phenylpropanoids, namely *trans*-anethole and estragole. In the same way, the *trans*-anethole relative content (*arc*) was also calculated. The obtained percentages were used as a measure of allele frequencies in the putative phenylpropanoid biosynthesis gene locus. Allele frequencies per population were calculated as the arithmetic averages obtained from single plant data. Gas chromatograms were also scored for the presence or absence of fenchone peak. The following criteria were used for fenchone scoring; score 2 (two fenchone alleles/genes): fenchone peak area > 15%; score 1 (single fenchone allele/gene): fenchone peak area < 15%; score 0 (no fenchone allele present): fenchone peak area = 0%. The sum of all scores was divided by the number of haploid genomes, in a given population, and used so as to calculate the fenchone presence allele frequency (*fp*) or complementally, fenchone absence allele frequency (*fa*).

### 3.3. PCR-RFLP

PCR amplification of the nrDNA ITS region was performed by using a TGradient thermal cycler (Whatman Biometra, Goettingen, Germany). The primers used [52] were ITS1 (5′-TCCGTAGGTGAACCTGCGG-3′) and ITS4 (5′-TCCTCCGCTTATTGATATGC-3′) (MWG-Biotech, Huntsville, AL, USA). Each PCR 25 μL reaction mixture consisted of 2.5 μL 10× PCR buffer (Promega, Fisher Scientific, Waltham, MA, USA), 0.8 mM dNTPs, 1 μM of each primer, 0.5 U of Taq polymerase (Promega) and 35 ng of template DNA. After the initial denaturation at 94 °C for 5 min, 35 PCR cycles were performed with 10 s at 93 °C, 10 s at 57 °C, and 45 s at 72 °C.

During preliminary tests, PCR products were subjected to endonuclease restriction by using several enzymes: *Alu*I, *Hae*III, *Mse*I, *Msp*I, *Rsa*I, *Sma*I, *Taq*I, and *Tsp*509I (New England Biolabs, Ipswich, MA, USA). For each endonuclease restriction reaction, 10 μL of the PCR product was incubated, for 16 h with 3 U of the corresponding enzyme, 1× appropriate buffer and 1× BSA, in a final volume of 15 μL, at the optimal enzyme incubating temperature. *Msp*I was finally selected for large-scale PCR-RFLP analyses. In this case, the restriction reactions were performed, using the abovementioned conditions, at 37 °C. Examples of PCR-RFLP products using *Msp*I are shown in Figure 2.

### 3.4. Gel Electrophoresis and Densitometry

Ten microliters of each PCR-RFLP product were loaded onto and separated in 1.5% agarose gels, containing 0.5 μg/mL of ethidium bromide, at 105 V for 1.75 h. Samples were bracketed (every 8 samples) with a 100 bp DNA size marker (Fermentas, Thermo Fisher Scientific, Waltham, MA, USA) as an aid in sizing the PCR-RFLP products. Gel visualization and image acquisition was performed under UV light. A Coolpix 8400 (Nikon, Tokyo, Japan) CCD camera was used to acquire gel images at the following settings: shutter speed 0.5 s, aperture F3.0, ISO setting 100, JPEG file format. The images were electronically filtered through a red channel filter and further used for analysis. For densitometric image analysis, Sorbfil TLC Videodensitometer software (Sorbpolymer, Krasnodar, Russian Federation) was used. Densitometry was performed on 684 bp, 529 bp, and 344 bp electrophoretic bands of PCR-RFLP products (Figure 2) and the band area percentages of these bands were used to estimate allele frequencies (*itsA*, *itsB*, and *itsC*, respectively) of the ITS locus in a sample. Population allele frequencies were calculated as arithmetic averages of single samples from a given population and used for data analysis.

### 3.5. Data Analysis

The allele frequencies calculated from genetic and chemical datasets (Table 1) were used to calculate Cavalli–Sforza and Edward’s chord distances [43] between populations. The obtained chord distances were used to reconstruct phenograms (Figure 1 and Figure 3) by means of the neighbor-joining method [45]. For chord distance calculations and phenograms reconstructions, NTSYSpc software version 2.02i (Applied Biostatistics, Setauket, NY, USA), was used (Tables S1 and S2).

## 4. Conclusions

Our findings indicate a high degree of genetic polymorphism and chemical variability both between and within fennel populations. The obtained gas chromatography data for phenylpropanoids and fenchone were employed for allele frequency estimation for their respective loci, in a similar manner as PCR-RFLP data. Population analyses were conducted on both chemical and genetic data separately, as well as in combination. The separate use of genetic and chemical data did not reveal an unambiguous relationship between both datasets, but the combined use of genetic and chemical information, due to their complementarity, proved to be useful in obtaining a more detailed and comprehensive insight into relationships between fennel populations in study, rather than the use of single data sets alone.

## Figures and Tables

**Table 1:** Maximum, arithmetic average and minimum values for obtained chemical and genetic data from fennel populations in study represented as putative allele frequencies. Data in parentheses are standard deviation values.

	Chemical Data					Genetic Data		
Locus	Fenchone			Phenylpropropanoids		nrDNA ITS		
Population	Allele	*fp*	*fa*	*erc*	*arc*	*itsA*	*itsB*	*itsC*
	max.	1.000	1.000	1.000	0.400	0.390	0.672	0.471
Ankaran	average	0.553 (0.28)	0.447 (0.28)	0.952 (0.12)	0.048 (0.12)	0.329 (0.02)	0.521 (0.12)	0.150 (0.13)
	min.	0.000	0.000	0.600	0.000	0.298	0.230	0.000
	max.	0.500	0.500	1.000	0.400	0.415	0.693	0.486
Buje	average	0.500 (0.00)	0.500 (0.00)	0.895 (0.12)	0.105 (0.12)	0.336 (0.05)	0.551 (0.12)	0.113 (0.16)
	min.	0.500	0.500	0.600	0.000	0.235	0.270	0.000
	max.	1.000	1.000	1.000	1.000	0.404	0.731	0.000
Flengi	average	0.526 (0.20)	0.474 (0.20)	0.597 (0.37)	0.403 (0.37)	0.352 (0.03)	0.648 (0.03)	0.000 (0.00)
	min.	0.000	0.000	0.000	0.000	0.269	0.596	0.000
	max.	0.500	1.000	1.000	0.969	0.374	0.691	0.230
Ližnjan	average	0.471 (0.12)	0.529 (0.12)	0.730 (0.28)	0.270 (0.28)	0.324 (0.03)	0.619 (0.07)	0.057 (0.09)
	min.	0.000	0.500	0.031	0.000	0.272	0.484	0.000
	max.	1.000	0.500	1.000	0.236	0.374	0.727	0.000
Padna	average	0.667 (0.25)	0.333 (0.25)	0.922 (0.12)	0.078 (0.12)	0.330 (0.03)	0.670 (0.03)	0.000 (0.00)
	min.	0.500	0.000	0.764	0.000	0.273	0.626	0.000
	max.	1.000	1.000	1.000	1.000	0.410	0.674	0.188
Plomin	average	0.474 (0.31)	0.526 (0.31)	0.589 (0.34)	0.411 (0.34)	0.359 (0.03)	0.594 (0.06)	0.047 (0.06)
	min.	0.000	0.000	0.000	0.000	0.307	0.505	0.000
	max.	1.000	0.500	0.036	1.000	0.449	0.702	0.409
Rabac	average	0.725 (0.26)	0.275 (0.26)	0.003 (0.01)	0.997 (0.01)	0.383 (0.05)	0.540 (0.08)	0.076 (0.11)
	min.	0.500	0.000	0.000	0.964	0.264	0.326	0.000
	max.	1.000	1.000	1.000	0.958	0.462	0.609	0.428
Rovinj	average	0.425 (0.29)	0.575 (0.29)	0.849 (0.23)	0.151 (0.23)	0.398 (0.06)	0.498 (0.09)	0.104 (0.14)
	min.	0.000	0.000	0.042	0.000	0.276	0.296	0.000
	max.	1.000	0.500	1.000	1.000	0.350	0.701	0.157
Vodnjan	average	0.700 (0.26)	0.300 (0.26)	0.798 (0.30)	0.202 (0.30)	0.321 (0.02)	0.634 (0.07)	0.044 (0.07)
	min.	0.500	0.000	0.000	0.000	0.297	0.544	0.000

*fp, fa,* fenchone presence and absence, respectively; calculated on the basis of fenchone % in essential oil; see Section 3.2. *erc, arc*,estragole and *trans-*anethole relative content, respectively; calculated as the percentage to the sum of both phenylpropanoids; see Section 3.2. *itsA*, *itsB*, *itsC*, relative band intensities of 684 bp, 529 bp and 344 bp electrophoretic bands, respectively; see Section 3.4.

**Table 2:** Replicate PCR-RFLP analyses (by using *Msp*I) on selected samples. The values are expressed as % of electrophoretic band intensity calculated on the sum of the three bands involved, the average of four replicates. Values in brackets are standard deviations of the measurements.

					Sample			
Band	Allele	1	2	3	4	5	6	7
684 bp	*itsA*	35.9 (2.9)	31.4 (1.4)	36.0 (3.4)	34.9 (0.8)	30.5 (2.3)	27.8 (2.4)	31.1 (2.1)
529 bp	*itsB*	64.1 (2.9)	68.6 (1.4)	56.7 (3.1)	50.4 (0.4)	49.3 (3.5)	48.9 (0.9)	24.2 (1.2)
344 bp	*itsC*	-	-	7.4 (0.3)	14.8 (0.6)	20.1 (1.4)	23.3 (1.4)	44.8 (2.0)

## Supplementary Materials

The following supporting information can be downloaded at: https://www.mdpi.com/article/10.3390/plants11172239/s1, Figure S1. Histograms for distribution of estragole and fenchone content in samples; Table S1. Matrix of chord distances for genetic data. Table S2. Matrix of chord distances for chemical data.
