# Supplementary material for: Chemical and Genetic Variability of Istrian Foeniculum vulgare Wild Populations
Source: Plants (Basel). 2022 Aug 29;11(17):2239. doi: 10.3390/plants11172239 (PMC9460853; doi:10.3390/plants11172239)
Supplement: Supplementary file 1 [file plants-11-02239-s001.zip › Figure S1.pdf]

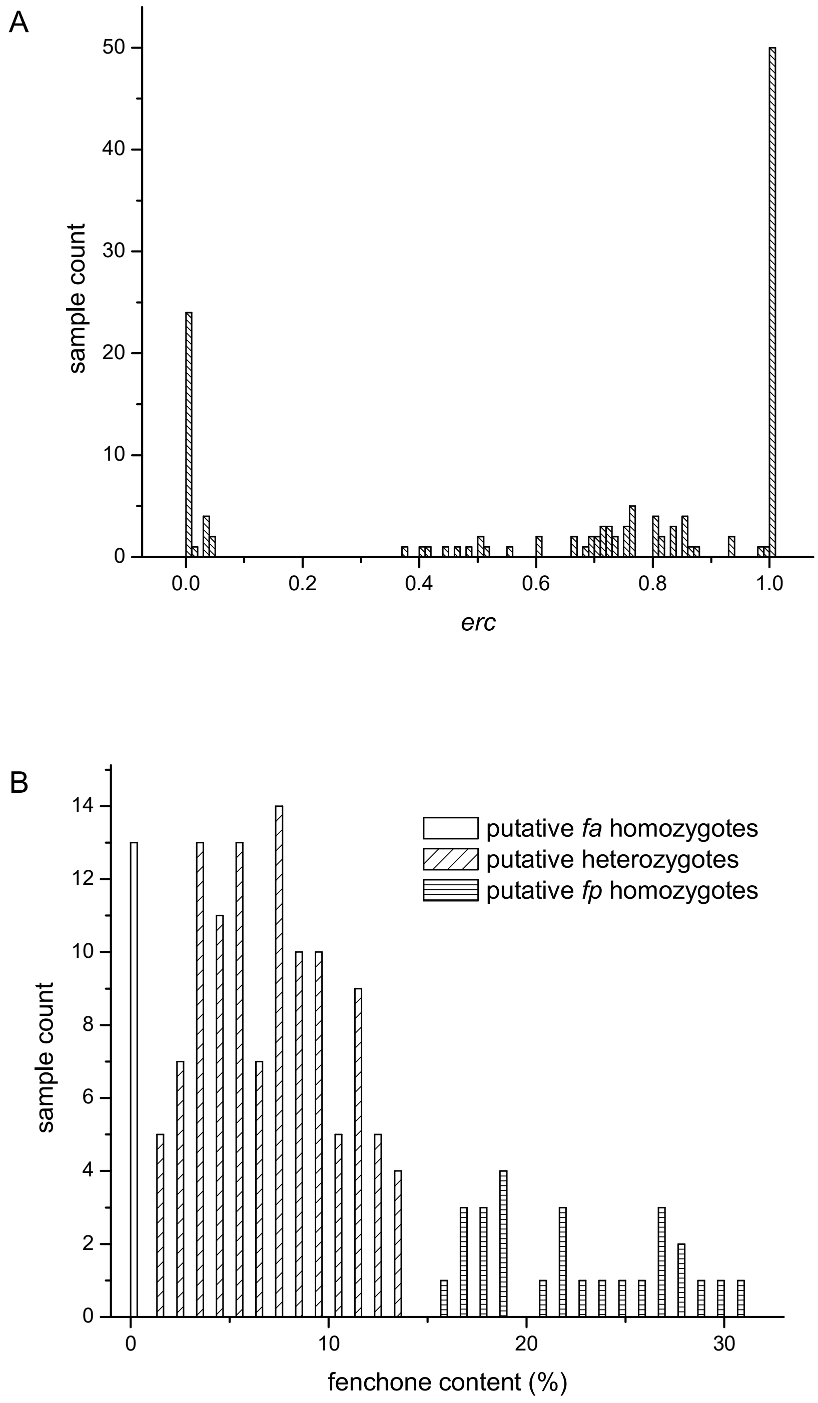

Figure S1. Histograms for distribution of (**A**) estragole relative content (*erc*) and (**B**) fenchone content in pooled samples. The results for fenchone content are represented as a distribution of 3 putative genetic groups.
