# Supplementary material for: Chemical and Genetic Variability of Istrian Foeniculum vulgare Wild Populations
Source: Plants (Basel). 2022 Aug 29;11(17):2239. doi: 10.3390/plants11172239 (PMC9460853; doi:10.3390/plants11172239)
Supplement: Supplementary file 1 [file plants-11-02239-s001.zip › Table S1.pdf]

Table S1. Matrix of chord distances for genetic data.

	**Ankaran**	**Buje**	**Flengi**	**Liznjan**	**Padna**	**Plomin**	**Rabac**	**Rovinj**	**Vodnjan**
Ankaran	0.0000								
Buje	−2.6770	0.0000							
Flengi	−1.3829	−1.4380	0.0000						
Liznjan	−2.5166	−2.4878	−1.5078	0.0000					
Padna	−1.3714	−1.4267	−1.5821	−1.4975	0.0000				
Plomin	−2.4869	−2.4618	−1.5355	−2.3608	−1.5240	0.0000			
Rabac	−2.6051	−2.5691	−1.5014	−2.4438	−1.4887	−2.4251	0.0000		
Rovinj	−2.6752	−2.6310	−1.4613	−2.4883	−1.4478	−2.4667	−2.5747	0.0000	
Vodnjan	−2.4547	−2.4313	−1.5227	−2.3348	−1.5127	−2.3185	−2.3937	−2.4324	0.0000
