# Supplementary material for: Chemical and Genetic Variability of Istrian Foeniculum vulgare Wild Populations
Source: Plants (Basel). 2022 Aug 29;11(17):2239. doi: 10.3390/plants11172239 (PMC9460853; doi:10.3390/plants11172239)
Supplement: Supplementary file 1 [file plants-11-02239-s001.zip › Table S2.pdf]

Table S2. Matrix of chord distances for chemical data.

	Ankaran	Buje	Flengi	Liznjan	Padna	Plomin	Rabac	Rovinj	Vodnjan
Ankaran	0.0000								
Buje	−2.5595	0.0000							
Flengi	−2.6150	−2.8902	0.0000						
Liznjan	−2.6478	−2.8934	−3.1999	0.0000					
Padna	−2.4626	−2.6115	−2.7297	−2.7332	0.0000				
Plomin	−2.6069	−2.8878	−3.2851	−3.2025	−2.7138	0.0000			
Rabac	−0.3190	−0.7007	−1.5317	−1.2500	−0.5399	−1.5263	0.0000		
Rovinj	−2.5919	−2.7988	−3.0010	−2.9916	−2.6447	−3.0051	−0.8651	0.0000	
Vodnjan	−2.5646	−2.7707	−3.0328	−2.9838	−2.6777	−3.0167	−1.0605	−2.8322	0.0000
